# Economic Evaluation of the Protecting Teeth @ 3 Randomized Controlled Trial

**DOI:** 10.1177/23800844221090444

**Published:** 2022-04-20

**Authors:** Y. Anopa, L.M.D. Macpherson, A.D. McMahon, W. Wright, D.I. Conway, E. McIntosh

**Affiliations:** 1 College of Medical, Veterinary and Life Sciences, Glasgow Dental School, University of Glasgow, Glasgow, UK; 2 Health Economics and Health Technology Assessment, Institute of Health and Wellbeing, University of Glasgow, Glasgow, UK

**Keywords:** dental caries, fluorides, cost-effectiveness analysis, cost-utility analysis, public health, child health

## Abstract

**Introduction:**

An economic evaluation (EE) was conducted alongside a randomized controlled trial (the Protecting Teeth @ 3 Study [PT@3]), exploring the additional preventive value of fluoride varnish (FV) application at 6-monthly intervals in nursery schools compared to treatment as usual (TAU) in the same nurseries. TAU represented a multicomponent national child oral health improvement intervention, the Childsmile program, apart from nursery FV.

**Methods:**

The EE was a within-trial cost-utility analysis (CUA) comparing the FV and TAU groups. The CUA was conducted from a National Health Service perspective and followed relevant methods guidance. Within-trial costs included intervention costs and health care resource use costs. Health outcomes were expressed in quality-adjusted life years (QALYs) accrued over the 2-y follow-up period. The Child Health Utility 9 Dimensions questionnaire was used to obtain utility scores. National reference costs were used, a discount rate of 1.5% for public health interventions was adopted, multiple imputation methods for missing data were employed, sensitivity analyses were conducted, and incremental cost-utility ratios were calculated.

**Results:**

Data from 534 participants from the 2014–2015 PT@3 intake were used in the EE analyses, n = 265 (50%) in the FV arm and n = 269 (50%) in the TAU arm. Mean incremental cost per child in the FV arm was £68.37 (P = 0.382; 95% confidence interval [CI], –£18.04 to £143.82). Mean incremental QALY was −0.004 (P = 0.636; 95% CI, −0.016 to 0.007). The probability that the FV intervention was cost-effective at the UK £20,000 threshold was 11.3%.

**Conclusion:**

The results indicate that applying FV in nurseries in addition to TAU (all other components of Childsmile, apart from nursery FV) would not be deemed cost-effective given current UK thresholds. In view of previously proven clinical effectiveness and economic worthiness of the universal nursery toothbrushing component of Childsmile, continuation of the additional, targeted nursery FV component in its pre–COVID-19 form should be reviewed given its low probability of cost-effectiveness.

**Knowledge Transfer Statement:**

The results of this study can be used by child oral health policy makers and dental public health professionals. They can form part of the evidence to inform the Scottish, UK, and international guidance on community-based child oral health promotion programs.

## Introduction

Dental caries is chronic in nature. It can affect very young children, and it is a lifelong condition that can continue across adolescence and adulthood and into later life (Peres et al. 2019). Dental caries is a preventable disease, and currently, a range of nationwide and community-based programs and clinical strategies exist to reduce caries prevalence in children. Notwithstanding the fact that childhood caries is widespread and that it poses a substantial economic burden (Phantumvanit et al. 2018; Righolt et al. 2018), there is a paucity of economic evaluations (EEs) of caries prevention interventions in preschoolers (Anopa et al. 2020). The lack of high-quality EEs makes it difficult for decision makers to determine which interventions to provide within the remit of health services and local authorities.

Childsmile is a Scotland-wide oral health improvement program for children. It comprises several components: daily supervised toothbrushing (with 1,000–1,450 ppm fluoride toothpaste) available in all nurseries (kindergartens) and in the first 2 y of primary school in the more socioeconomically deprived areas, distribution of free toothpaste and toothbrush packs for home use, community-based dental health support worker home visits, biannual applications of fluoride varnish (FV) in targeted nurseries and primary schools, and preventive care, including FV and oral health advice within primary care dental services (Macpherson et al. 2019a, 2019b). One component of Childsmile targeted at children at an increased risk of dental caries is a nursery- and school-based FV application scheme. Children in the most deprived areas in each National Health Service (NHS) Health Board (administrative area) in Scotland are offered twice-yearly application of FV via the education setting.

The universal nursery supervised toothbrushing component of Childsmile has previously been shown to be both effective and cost saving (Macpherson et al. 2013; Anopa et al. 2015), but there has been no EE of the additional nursery-based twice-yearly FV application component of Childsmile. A recent systematic review of EEs in preschoolers’ caries prevention (Anopa et al. 2020) has identified only 2 studies on FV that employed cost-effectiveness analysis (CEA) (Quinonez et al. 2006; O’Neill et al. 2017) and did not find any EEs conducted alongside caries prevention randomized controlled trials undertaken in nursery settings.

The nursery-based Protecting Teeth @ 3 (PT@3) trial aimed to assess the effectiveness and cost-effectiveness of additional preventive twice-yearly FV application plus other Childsmile program interventions as usual, compared to usual Childsmile interventions alone (treatment as usual [TAU]) (Wright et al. 2015; McMahon et al. 2020). The effectiveness results of the PT@3 study showed a modest and nonsignificant anticaries effect (McMahon et al. 2020).

The aim of the EE conducted alongside the trial was to estimate the cost-effectiveness of the FV plus TAU intervention compared with TAU only (control) in 3 ways: 1) to conduct a cost-utility analysis (CUA) comparing the costs and utilities of the 2 groups over a 24-mo period, 2) to conduct a CEA comparing costs and effects between groups (oral health improvement or worsening, as measured by the number of decayed, missing and filled teeth [d3mft], and 3) to conduct a cost-consequence analysis (CCA) including all available costs and outcome measures: the results of the CUA and CEA, as well as other general health–related quality of life (GHQoL) and oral health–related quality of life (OHQoL) measures.

The full protocol and the clinical outcomes of the PT@3 trial are presented elsewhere (Wright et al. 2015; McMahon et al. 2020).

## Methods

The PT@3 study was a parallel-group randomized controlled trial with a 2-y follow-up. Ethical approval was obtained from the NHS West of Scotland Research Ethics Committee (12/WS/0136). The participants were 3-y-old children attending nursery schools. Written informed consent was obtained from the parents or guardians of the children. The children were randomized (1:1) to the intervention group (FV plus TAU, referred to as “FV” group from here on) or the control group (receiving TAU only). Children in the intervention group had Duraphat (Colgate) FV (50 mg/mL) applied to the surfaces of the primary teeth and also continued to receive TAU: all other components of Childsmile (children attended their usual sources of dental care during the trial and dental practitioners continued with their normal care; the children also received the other Childsmile interventions, regardless of their treatment allocation) (McMahon et al. 2020). Children in the TAU group received the same series of contacts, with a “mock” FV application (with an applicator brushing the teeth with no FV on it) and continued with TAU. Interventions were undertaken by Childsmile-trained extended-duty dental nurses at 6-monthly intervals, with a total maximum number of 4 FV applications over the 2-y course of the study. Participants received a baseline dental inspection in nursery at the age of 3 y and an end-point inspection in the first year of primary school at the age of 5 y by trained and calibrated examiners (Wright et al. 2015; McMahon et al. 2020). The schedule of contacts with the participating children is summarized in Appendix Figure 1. This trial was registered at EUDRACT (2012-002287-26) and ClinicalTrials.gov (NCT01674933).

In PT@3, the nurseries that were targeted in each NHS Health Board area were those just above the cutoff for inclusion in the FV scheme for the main Childsmile program, namely, the next most socially disadvantaged areas based on the Scottish Index of Multiple Deprivation (SIMD) (Scottish Government 2012) of the home postcode of the children. The overall study was conducted in 4 NHS Health Boards in Scotland: Greater Glasgow and Clyde, Fife, Lothian, and Tayside; only the latter 3 NHS Health Boards participated in the EE segment of the trial. The EE was based on the children who were recruited into the study within the 2014–2015 academic year.

A number of outcomes were measured. The primary clinical effectiveness outcome was dental caries as measured by d3mft, which was used in CEA. The EE outcome measures also included 4 different GHQoL and OHQoL measures collected at 3 points in time: the start of study (baseline), midstudy (in 12 mo), and end of study (in 24 mo). The GHQoL measures were the Child Health Utility 9 Dimensions (CHU9D) (Stevens 2009, 2011, 2012) and the Paediatric Quality of Life (PedsQL) Core, toddler version (Buck 2012; Varni 2020), while the OHQoL measures were the PedsQL Oral Health module (PedsQL-OH) (Steele et al. 2009) and the Scale of Oral Health Outcomes for 5-Year-Old Children (SOHO-5) (Tsakos et al. 2012; Abanto et al. 2013) (Appendix Table 1 and Appendix Figure 2). Parental proxy-reported versions were used for all instruments. The reference period used was “prior 12 mo.”

### Within-Trial Economic Analysis Methods

Following the United Kingdom’s National Institute for Health and Care Excellence (NICE) public health economic evaluation guidelines, a public-sector perspective was taken, that of the UK’s NHS, and a discount rate of 1.5% was employed (NICE 2012). The time horizon was the follow-up of the PT@3 study: 2 y. The year of study completion, 2016–2017, was used as the cost baseline year. All costs were valued in UK pounds sterling (£).

Three types of EE analyses were conducted: CUA, CEA, and CCA. A brief description of each type of analysis is presented in Appendix Box 1.

### Costs

A micro-costing (bottom-up) approach was used to estimate the costs. Resource use was identified through discussions with the trial managers and coordinators, based on previous EEs of child dental health trials (Tickle et al. 2016; Chestnutt et al. 2017) and by conducting observational visits to nurseries participating in the PT@3 trial.

Resource use was measured over the duration of the trial and was made up of the following data collection:

1. Intervention costs (for the FV group), which consisted of staff labor, staff travel costs, and the costs of disposable and reusable materials

Labor and staff travel costs were collected during 2014–2015 to 2016–2017 (from the time the participants entered the trial to the time of their final interventions) by using a time and travel data capture form (Appendix Fig. 3). The form contained the information on the names of the staff involved in a visit, mileage to and from the nursery, and the duration of the visit.

The cost of travel was calculated as mileage related to each nursery visit multiplied by the mileage rate. The rate used was 47.5 p per mile (HM Revenue and Customs 2012), based on an average number of PT@3 staff traveling together in one vehicle, which was 1.5 persons per car/van.

NHS pay bands for each of the PT@3 staff members were requested from trial coordinators, and midpoint salaries for each respective band range were used in the calculations (Royal College of Nursing 2016). The information on costs of disposable and reusable items used during FV visits was requested from the trial coordinators.

2. Participant health care (NHS) resource use, including service use and medications (for children in both study groups). It was collected within trial with the help of a resource use questionnaire at baseline, 12 mo, and 24 mo. At each time point, the respondents were asked about their child’s health care resource use within the preceding 12 mo. The number of contacts with the following health services was recorded: general practitioner (GP), Accident and Emergency (A&E), family dentist, dental hygienist, speech and language therapist, hospital inpatient and outpatient stay, and any other health care services used (free text) (see Appendix Fig. 2).

Unit cost information was identified from routine sources such as the Personal Social Services Resource Unit (PSSRU) (Curtis and Burns 2017) and the NHS Reference Costs (NHS Improvement 2017). See Appendix Table 2 for resource use unit costs.

3. Family costs (representing societal costs), which included time away from work/usual activities due to child’s ill health, as shown in Appendix Table 3. Family costs were included in a sensitivity analysis.

The methods for handling missing data are described in Appendix Box 2.

### Cost-Utility Analysis

The mean costs and quality-adjusted life years (QALYs) for each group were presented using the method of recycled predictions (Glick et al. 2014). Differences in cost and QALYs between the intervention and control groups were estimated using generalized linear models (Barber and Thompson 2004). Costs and QALYs were adjusted for the following baseline covariates: treatment group, age, sex, SIMD, caries at baseline, baseline utility, and baseline resource use cost. Analyses were conducted in Stata version 16.0 (StataCorp).

### Probabilistic Sensitivity Analysis

A probabilistic sensitivity analysis was conducted by jointly bootstrapping the mean difference in cost and QALYs to produce 1,000 paired estimates. Nonparametric bootstrap with replacement was used (Glick et al. 2014). The results of the bootstrapping were graphically presented on a cost-effectiveness plane, and a cost-effectiveness acceptability curve was constructed. In the United Kingdom, interventions are considered cost-effective if the cost per additional QALY gained is within the range of £20,000 to £30,000 per QALY gained (NICE 2013).

### Cost-Utility Sensitivity Analyses

Several 1-way sensitivity analyses were designed a priori in order to assess the impact of uncertainty on the cost-effectiveness results (Appendix Table 4).

### Other Types of Analyses

CEA and CCA methods are described in Appendix Tables 5 and 6.

The reporting of this study complies with the CHEERS checklist (Husereau et al. 2013) (see Appendix Table 7).

## Results

The data for 534 participants from the 2014–2015 PT@3 intake were used in the EE analyses. The baseline characteristics of the EE sample are described in Appendix Table 8. With regards to intervention costs in the FV group, the major component of the average cost per child per visit was staff labor, which accounted for 64% (£7.59), followed by disposables (20%, £2.32), staff travel (15%, £1.78), and reusables (1%, £0.08). Materials costs for the FV group are detailed in Appendix Table 9.

The mean (SD) intervention cost per child in the FV group over the whole course of the study was £32.66 (£13.21).

The intervention (FV plus TAU) was found to be dominated by the comparator (TAU only). The intervention group had slightly worse outcomes and cost more than TAU only, although all differences in total costs and outcomes between the groups were not statistically significant. This held true in both cases: when the outcome was QALYs (Table 1) and when the outcome was the change in d3mft (Table 2). The CUA results showed that compared to the control group, the intervention group had an incremental cost of £68.37 (95% confidence interval [CI], –£18.04 to £143.82; *P* = 0.382) and a marginal incremental QALY loss: −0.0044 (95% CI, −0.016, 0.0069; *P* = 0.636). This result was also persistent in all CUA sensitivity analysis scenarios (Appendix Table 10).

**Table 1.** Cost-Utility Results (after Imputation) with 24-mo Follow-up.

Treatment Group	Cost (£)			QALY		
	Mean	SE	95% CI	Mean	SE	95% CI
FV (intervention)	665.90	70.74	564.38 to 752.84	1.8590	0.0078	1.8483 to 1.8674
TAU (control)	597.52	70.67	519.29 to 674.27	1.8634	0.0074	1.8522 to 1.8729
Difference	68.37	*P* = 0.382	−18.04 to 143.82	−0.0044	*P* = 0.636	−0.016 to 0.0069
ICER	Dominated^a^					

The total cost per participant includes the total cost of health care resources used over the 2-y duration of the study but excludes “other” resource use items—for both groups and also the intervention group (FV), total cost includes total intervention cost. General linear modeling was used. Both cost and QALY were adjusted for sex, age, deprivation, baseline utility, and caries at baseline. Second-year costs and QALYs were discounted at a 1.5% discount rate.

CI, confidence interval; FV, fluoride varnish; ICER, incremental cost-effectiveness ratio; QALY, quality-adjusted life year; TAU, treatment as usual.

a With very small numerical differences in effect in favor of TAU and TAU being less costly than FV, the ICER is calculated as dominated. However, this calculation is based on nonstatistically significant differences in outcomes between FV and TAU.

**Table 2.** Cost-Effectiveness Results on d3mft, with 24-mo Follow-up.

Treatment Group	Cost (£)			d3mft Difference (24 mo – 0 mo)		
	Mean	SE	95% CI	Mean	SE	95% CI
FV (intervention)	665.90	70.74	564.38 to 752.84	0.992	0.118	0.761 to 1.239
TAU (control)	597.52	70.67	519.29 to 674.27	0.921	0.118	0.695 to 1.148
Difference	68.37	*P* = 0.382	−18.04 to 143.82	0.071	*P* = 0.671	−0.237 to 0.406
ICER	Dominated^a^					

The total cost per participant includes the total cost of health care resources used over the 2-y duration of the study but excludes “other” resource use items—for both groups and also the intervention group (FV), total cost includes total intervention cost. General linear modeling was used. Both cost and d3mft difference were adjusted for sex, age, deprivation, baseline utility, and caries at baseline. Cost analysis was conducted on a multiple-imputed data set (*n* = 534). d3mft was analyzed on a complete case data set (*n* = 508 [95%] out of 534). Second-year costs were discounted at a 1.5% discount rate.

CI, confidence interval; d3mft, number of decayed, missing and filled teeth; FV, fluoride varnish; ICER, incremental cost-effectiveness ratio; TAU, treatment as usual.

a With small numerical differences in effect in favor of TAU and TAU being less costly than FV, the ICER is calculated as dominated. However, this calculation is based on nonstatistically significant differences in outcomes between FV and TAU.

**Figure 1.**
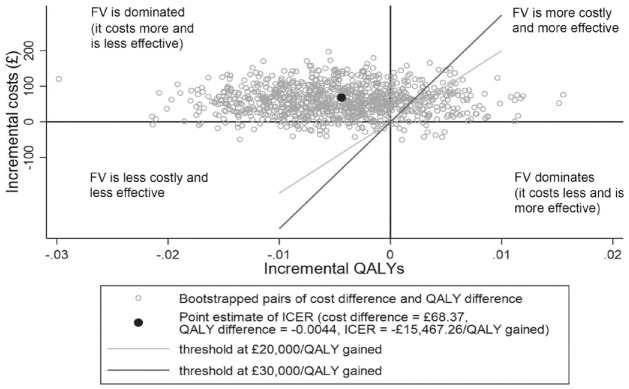
Cost-effectiveness plane representing 1,000 bootstrapped cost difference and QALY difference pairs. FV, fluoride varnish (intervention group); ICER, incremental cost-effectiveness ratio; QALY, quality-adjusted life year.

The cost-effectiveness plane for CUA base-case analysis is shown in Figure 1. The fact that most dyads are situated in the northwest quadrant means that in the majority of the bootstrap iterations, TAU dominates: namely, the FV intervention is less effective (in terms of QALY gained) and more costly than TAU.

**Figure 2.**
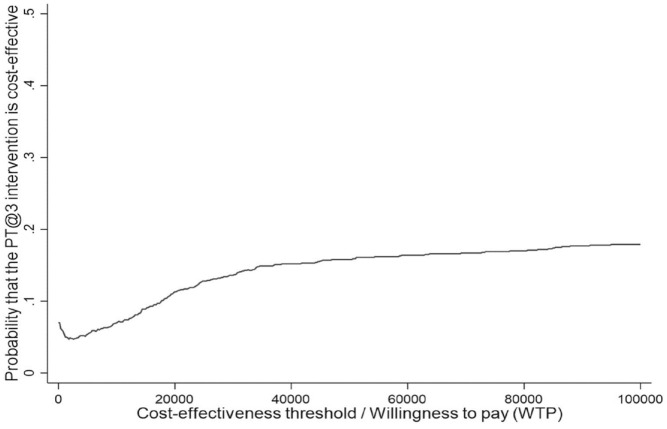
Cost-effectiveness acceptability curve.

Figure 2 shows the cost-effectiveness acceptability curve. It indicates that there would be an 11.3% probability of the FV intervention being cost-effective at a NICE societal willingness-to-pay threshold of £20,000 per additional QALY. In all considered sensitivity analysis scenarios, this probability was also low (9.2% to 19.8%), as shown in Appendix Table 10.

**Figure 3.**
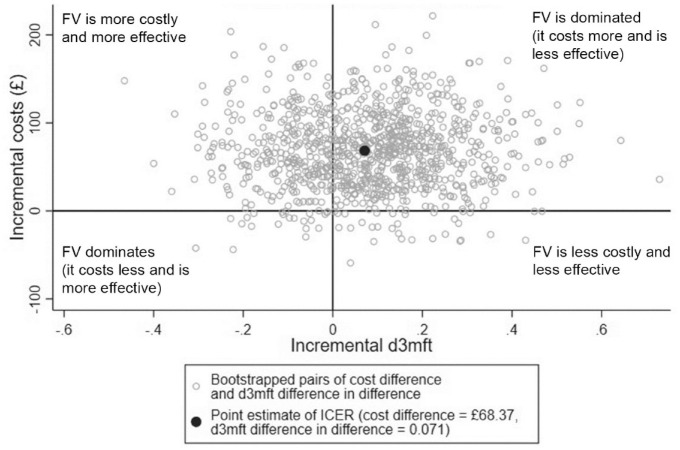
Cost-effectiveness plane representing 1,000 bootstrapped cost difference and d3mft difference in difference pairs. Positive values along the x-axis (incremental d3mft) mean the worsening of the dental outcome; therefore, the quadrants differ from the usual quadrant representation. Here, northwest quadrant = FV is more costly and more effective; northeast = FV is dominated (it costs more and is less effective); southeast = FV is less costly and less effective; and southwest = FV dominates (it costs less and is more effective). d3mft, number of decayed, missing and filled teeth; FV, fluoride varnish (intervention group); ICER, incremental cost-effectiveness ratio.

The results of the CEA showed that the mean d3mft difference was slightly higher in the intervention group (meaning greater oral health worsening), in comparison with the TAU group, indicating that on average, the intervention arm children had a slightly greater worsening of the d3mft (Table 2). However, the difference in difference between the groups was only 0.071 and was not statistically significant. The results of the probabilistic sensitivity analysis with the d3mft difference in difference as an effectiveness measure showed that in 66% of the 1,000 bootstrap simulations, the intervention was more costly and less effective, while in 29%, the intervention was more costly and more effective (Figure 3).

The results of the CCA indicated that none of the total cost or outcome differences between the 2 groups were statistically significant (Appendix Table 11).

## Discussion

The universal nursery toothbrushing component of Childsmile was previously shown to be both clinically effective (Macpherson et al. 2013) and highly cost-saving. It was most cost-saving in children from the most socioeconomically deprived areas (Anopa et al. 2015). Moreover, a recent Childsmile data linkage study, which aimed to evaluate the reach of the program and the impact of its components on child oral health (Kidd et al. 2020), indicated that compared to those children who did not participate in the nursery-supervised toothbrushing intervention, there was a reduction in the odds of caries experience as the number of years of participation in toothbrushing increased. Kidd et al. (2020) also indicated that odds of caries experience were markedly lower among children regularly attending Childsmile appointments at dental practice. However, children receiving FV at nursery, in comparison to children receiving zero applications, had no reduction in the odds of caries experience regardless of the number applied.

Only 2 previous randomized controlled trials that investigated the effectiveness and cost-effectiveness of FV in 1- to 5-y-old children were identified (O’Neill et al. 2017; Anderson et al. 2019). A large Northern Ireland trial was undertaken in dental practices with children 2 to 3 y of age at baseline and followed up for 2 y (Tickle et al. 2016; O’Neill et al. 2017). A nonsignificant marginal benefit of FV compared to preventive advice only was found. Their results showed that the intervention was potentially cost-effective only with respect to reducing carious surfaces (but not for the proportion of children who remained caries free or for the number of episodes of pain). A Swedish trial evaluated an enhanced caries-preventive program, which included FV, in comparison with the standard preventive program in children initially aged 12 mo and followed up for 2 y (Anderson et al. 2019). This trial was somewhat similar to the PT@3 study, as their standard program already comprised many preventive efforts. The additional intervention was FV and a higher frequency of the other interventions. No significant difference in caries prevalence or number of decayed, extraction needed and filled tooth surfaces (defs) between the trial groups was found, and the enhanced program was not cost-effective. The results of these studies as well as the results of the PT@3 EE indicate that the overall cost-effectiveness of FV when compared to other interventions (as opposed to a “do nothing”/no intervention comparator) is questionable.

No difference between the study groups in the PT@3 study was found with regard to any of the GHQoL/OHQoL measures at 24 mo. Research shows that even in populations of children with dental caries, there is a wide variation in impacts that children can experience, with many of them displaying no symptoms at all (Tickle et al. 2002; Rogers et al. 2020). Hence, even oral health–specific quality-of-life instruments may not pick up any substantial signals. The PT@3 population was a combination of children with no obvious caries and children with comparatively low d3mft scores, with around 30% of children having caries at the end of the study (unlike, for example, populations recruited from dental practices, who are already known to require dental treatment); therefore, they were more likely not to exhibit any oral health– and general health–related symptoms. A previous study found CHU9D, the only available child-centered generic preference-based instrument, to be unresponsive to changes in the number of decayed, missing and filled surfaces in deciduous and permanent teeth (dmfs + DMFS) index score following caries treatment (Foster Page et al. 2015). It has been suggested that CHU9D might not be sensitive enough to be used as an outcome measure in EE in the area of pediatric dentistry (Foster Page et al. 2015) and that further psychometric testing of this measure is required to fully assess its suitability for use in longitudinal studies (Knapp 2019).

There are several limitations of the EE of the PT@3 study. The EE was conducted on a relatively small sample, which did not allow for meaningful subgroup analyses (by deprivation categories or by presence/absence of caries at baseline), as had been initially planned. The time horizon was the duration of the PT@3 trial, namely, 24 mo, hence, the EE results do not reflect likely longer-term cost-effectiveness throughout later childhood or over the whole life course.

One of the strengths of the PT@3 EE was that a bottom-up approach was used in the data collection and calculation of costs and outcomes. This EE has been undertaken alongside a rigorous randomized controlled trial, and data were collected prospectively. This approach allowed for a more precise estimation of both costs and outcomes, in comparison with, for example, using assumptions and/or previously published information. In addition, multiple outcomes were measured in the PT@3 study. These included clinical outcomes and several quality-of-life measures: a preference-based GHQoL measure (CHU9D), which allowed calculation of QALYs; a widely used non-preference-based GHQoL measure (PedsQL Core); and 2 OHQoL measures (PedsQL-OH and SOHO-5).

## Conclusions

The EE results show that applying FV in nursery settings in addition to the existing TAU (which was all other components of the Childsmile program, apart from nursery FV) is not likely to be cost-effective given current thresholds. In view of previously proven clinical effectiveness and economic worthiness of the universal nursery toothbrushing component of Childsmile, which was shown to be highly cost saving, it seems that the continuation of the targeted nursery FV program in its most recent (pre–COVID-19) form and shape in addition to nursery toothbrushing and other routine Childsmile components should be reviewed in consultation with policy makers. The results should form part of the evidence to inform the Scottish, UK, and international guidance on community-based child oral health promotion programs.

## Author Contributions

Y. Anopa, contributed to conception, design, data acquisition, analysis, and interpretation, drafted and critically revised the manuscript; L.M.D. Macpherson, D.I. Conway, contributed to conception, design, and data interpretation, critically revised the manuscript; A.D. McMahon, E. McIntosh, contributed to conception, design, data analysis, and interpretation, critically revised the manuscript; W. Wright, contributed to conception, design, and data acquisition, critically revised the manuscript. All authors gave final approval and agree to be accountable for all aspects of the work.

## Supplemental Material

sj-docx-1-jct-10.1177_23800844221090444 – Supplemental material for Economic Evaluation of the Protecting Teeth @ 3 Randomized Controlled Trial Click here for additional data file. Supplemental material, sj-docx-1-jct-10.1177_23800844221090444 for Economic Evaluation of the Protecting Teeth @ 3 Randomized Controlled Trial by Y. Anopa, L.M.D. Macpherson, A.D. McMahon, W. Wright, D.I. Conway and E. McIntosh in JDR Clinical & Translational Research
