# Supplementary material for: Economic Evaluation of the Protecting Teeth @ 3 Randomized Controlled Trial
Source: JDR Clin Trans Res. 2022 Apr 20;8(3):207–14. doi: 10.1177/23800844221090444 (PMC10285425; doi:10.1177/23800844221090444)
Supplement: sj-docx-1-jct-10.1177_23800844221090444 – Supplemental material for Economic Evaluation of the Protecting Teeth @ 3 Randomized Controlled Trial [file sj-docx-1-jct-10.1177_23800844221090444.docx]

**APPENDIX**

**Economic Evaluation of the Protecting Teeth @ 3 Randomised Controlled Trial**

Anopa Y., Macpherson L.M.D., McMahon A.D., Wright W., Conway D.I., McIntosh E.

Appendix Figure 1: Trial visit schedule

**Consent &**

**Contraindication Screening**

**Baseline Dental Inspection & Prescription of Duraphat^®^**

**Randomisation**

**Intervention**

**(Fluoride Varnish & TAU)**

**Control**

**(Treatment as usual, TAU)**

**Month 0 (baseline)**

**Intervention**

**(Fluoride Varnish & TAU)**

**Control**

**(TAU)**

**Month 6**

**Intervention**

**(Fluoride Varnish & TAU)**

**Control**

**(TAU)**

**Month 12**

**Intervention**

**(Fluoride Varnish & TAU)**

**Control**

**(TAU)**

**Month 18**

**Intervention**

**Endpoint Dental Inspection**

**Control**

**Endpoint Dental Inspection**

**Month 24 (endpoint)**

**Inclusion / Exclusion criteria applied immediately prior to inspection**

*Contraindication Screening* *Update*

*Contraindication Screening* *Update*

*Contraindication Screening* *Update*

Appendix Table 1: Economic evaluation outcome measures

| **Outcome measure** | **Baseline** | **12 mo.** | **24 mo.** | **Respondent / Source** | **Measuring what?** | **Economic**  **evaluation**  **framework** |
| --- | --- | --- | --- | --- | --- | --- |
| **d3mft** | X |  | X | PT@3 dental inspection | Clinical effectiveness | CEA / CCA |
| **CHU9D**  (A preference-based measure, allowing to calculate utility and QALYs) | X | X | X | Parent/carer | GHQoL | CUA / CCA |
| **PedsQL** | X | X | X | Parent/carer | GHQoL | CCA |
| **PedsQL-OH** | X | X | X | Parent/carer | OHQoL | CCA |
| **SOHO-5** | X | X | X | Parent/carer | OHQoL | CCA |

Notes: CHU9D – Child Health Utility 9 Dimensions, PedsQL – Paediatric Quality of Life Core, PedsQL-OH – PedsQL Oral Health module, SOHO-5 – Scale of Oral Health Outcomes for 5-year-old Children; d3mft - the number of decayed (into dentine), missing and filled teeth; QALY – quality adjusted life year; GHQoL – general health related quality of life, OHQoL – oral health related quality of life; CUA – cost-utility analysis, CEA – cost-effectiveness analysis; CCA – cost-consequence analysis; mo. – months.

Appendix Figure 2: Parental questionnaire


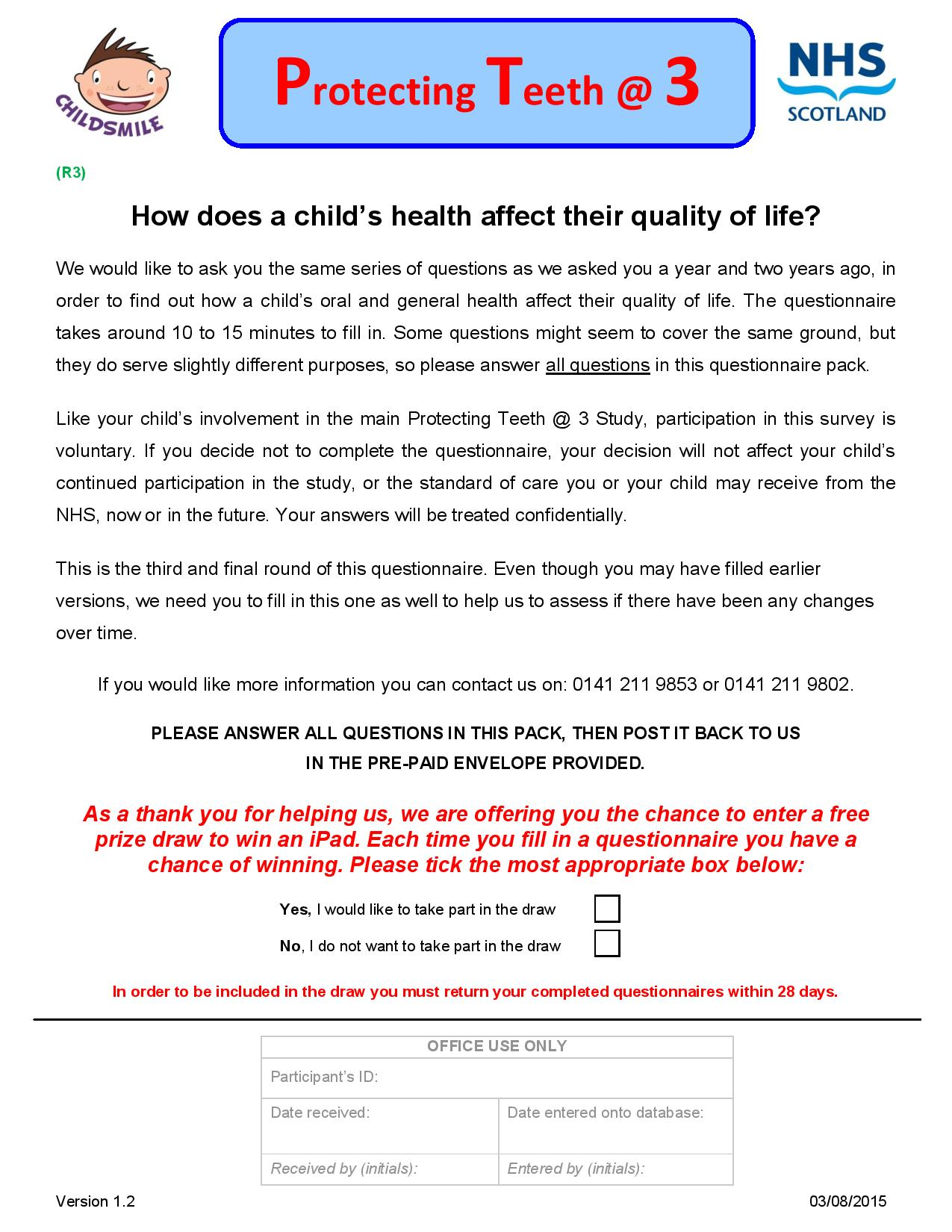


Three of the four of the health-related quality of life questionnaires used in this study are not presented in full due to restricted use. They can only be used under license.

**Child Health Utility 9 Dimensions (CHU9D)**

The CHU9D is a paediatric generic preference-based measure of health-related quality of life. It consists of a short questionnaire and a set of preference weights using general population values. The questionnaire has nine questions with five response levels per question. It is proxy completed for younger children (and self-completed for older children).

The CHU9D allows the analyst to obtain quality adjusted life years (QALYs) directly for use in cost-utility analysis.

Further details regarding the licencing of the CHU9D questionnaire and additional information can be found here: <https://licensing.sheffield.ac.uk/product/CHU-9D>

A preschooler parental proxy questionnaire version of the CHU9D was used in the Protecting Teeth @ 3 Study. The questionnaire has nine dimensions:

1. Worried
2. Sad
3. Pain
4. Tired
5. Annoyed
6. Nursery or school tasks / homework
7. Sleep
8. Daily routine
9. Ability to join in activities

There are five response levels per question. For example, the possible responses for the “Worried” question are as follows:

- My child doesn’t feel worried today
- My child feels a little bit worried today
- My child feels a bit worried today
- My child feels quite worried today
- My child feels very worried today

A sample self-complete questionnaire for an older child can be found here: <https://www.sheffield.ac.uk/polopoly_fs/1.44111!/file/Health-Questionnaire-final-watermarked.pdf> (However, note that a parental proxy version for preschoolers was used in this study.)

**PedsQL Generic Core Scales, Paediatric Quality of Life Inventory (parent report for toddlers aged 2-4 years)**

PedsQL Generic Core Scales questionnaire has four domains. Each domain consists of several questions (ranging from three to eight questions per domain).

PHYSICAL FUNCTIONING (problems with…):

1. Walking

2. Running

3. Participating in active play or exercise

4. Lifting something heavy

5. Bathing

6. Helping to pick up his or her toys

7. Having hurts or aches

8. Low energy level

EMOTIONAL FUNCTIONING (problems with…)

1. Feeling afraid or scared

2. Feeling sad or blue

3. Feeling angry

4. Trouble sleeping

5. Worrying

SOCIAL FUNCTIONING (problems with…)

1. Playing with other children

2. Other kids not wanting to play with him or her

3. Getting teased by other children

4. Not able to do things that other children his or her age can do

5. Keeping up when playing with other children

SCHOOL FUNCTIONING (problems with…)

1. Doing the same school activities as peers

2. Missing school/day-care because of not feeling well

3. Missing school/day-care to go to the doctor or hospital

There are five response levels per question:

0 = never a problem

1 = almost never a problem

2 = sometimes a problem

3 = often a problem

4 = almost always a problem

A sample copy of the PedsQL Core parent report for toddlers (ages 2-4) can be found on page 22 here: <https://eprovide.mapi-trust.org/instruments/pediatric-quality-of-life-inventory/rc_pedsql-4.0-core-all_au4.0_eng-usori>

More information on the PedsQL Paediatric Quality of Life Inventory can be found here: <http://www.pedsql.org/about_pedsql.html>

The conditions for use of the PedsQL scales can be found here: <http://www.pedsql.org/conditions.html>

**PedsQL Oral Health (parent report for toddlers aged 2-4 years)**

PedsQL Oral Health is a short add-on module that can be used together with the PedsQL Core questionnaire. There are five questions in the PedsQL Oral Health module.

ABOUT MY CHILD’S TEETH AND MOUTH (problems with…)

1. Having tooth pain

2. Having tooth pain when eating or drinking something hot, cold, or sweet

3. Having teeth that are dark in colour

4. Having gum pain

5. Having blood on his or her toothbrush after brushing

There are five response levels per question (same as for PedsQL Core):

0 = never a problem

1 = almost never a problem

2 = sometimes a problem

3 = often a problem

4 = almost always a problem

A sample copy of the PedsQL Oral Health parent report for toddlers (ages 2-4) can be found on page 14 here: <https://eprovide.mapi-trust.org/instruments/pediatric-quality-of-life-inventory/rc_pedsql-3.0-oralhealth-all_au3.0_eng-usori>


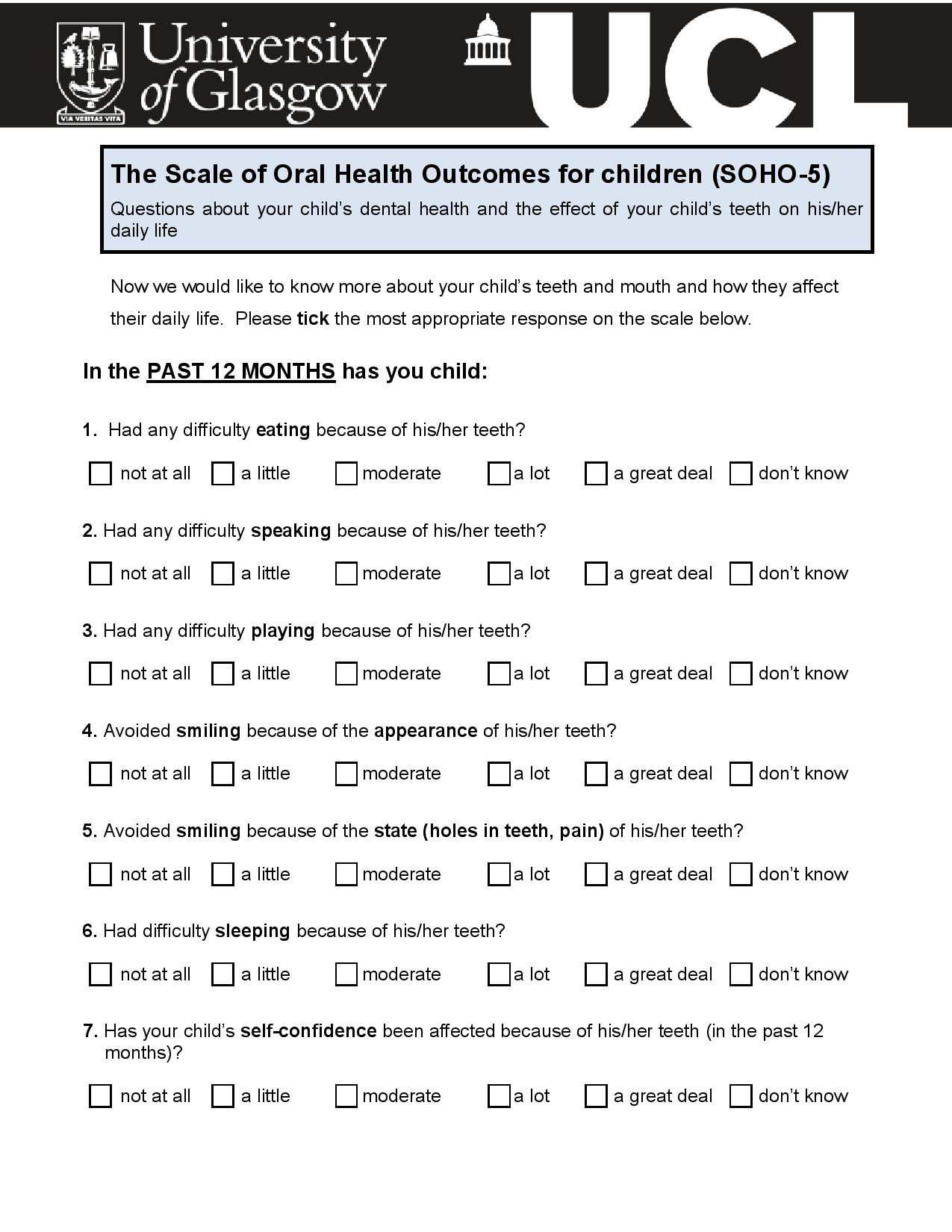


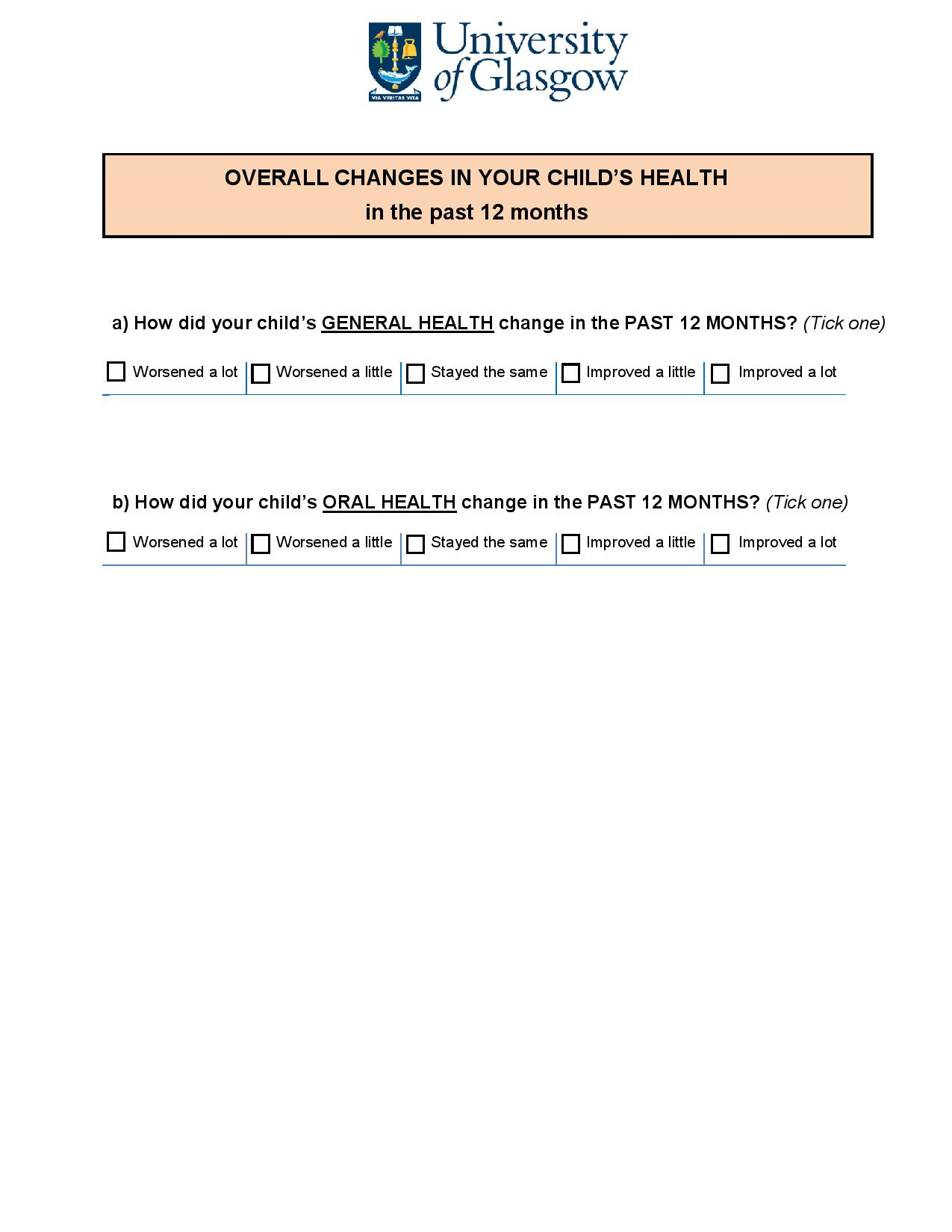


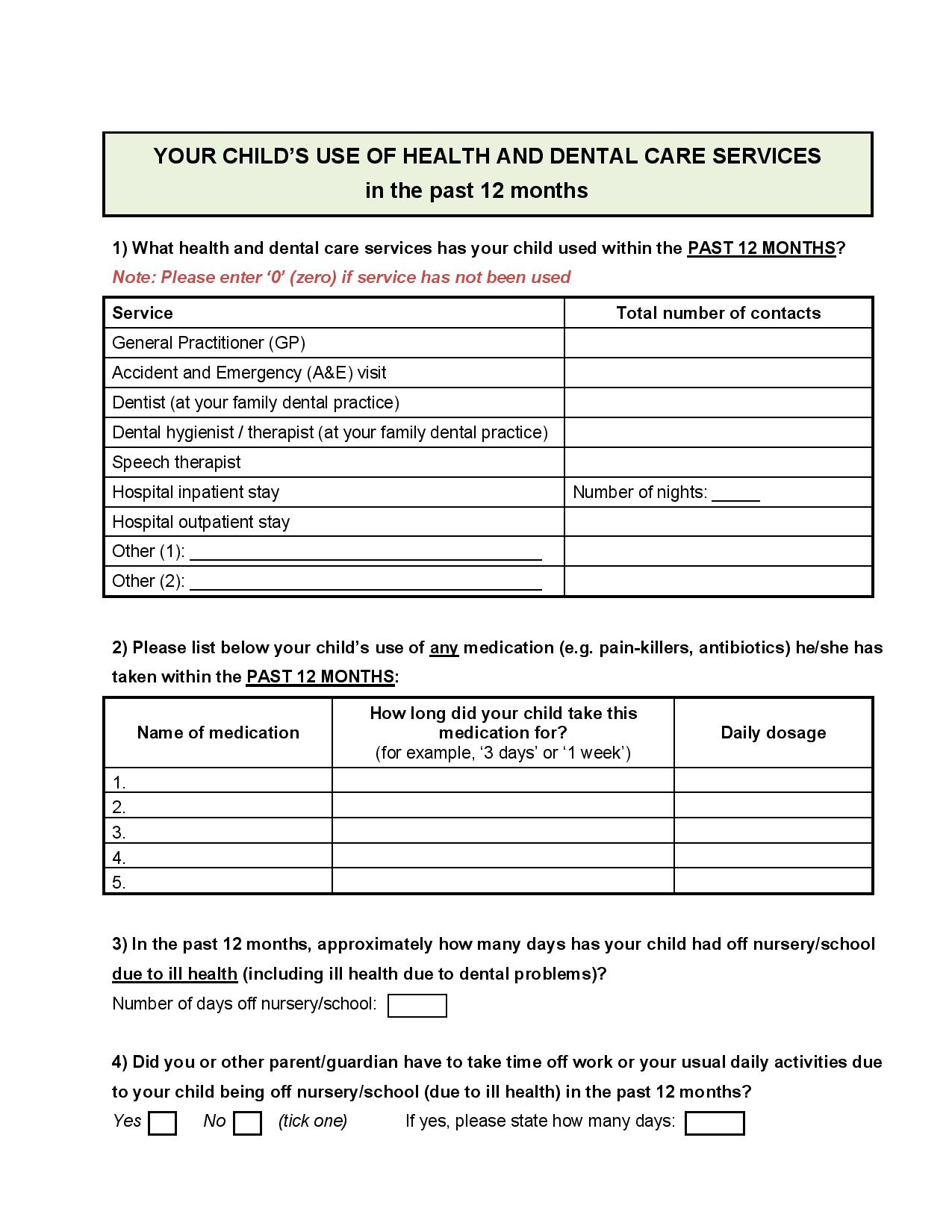


Appendix Box 1: Types of economic evaluation analyses used

| Cost-utility analysis (CUA) is an EE in which the effects of different interventions are measured using utility units (e.g. QALYs). Alternative interventions are then compared in terms of incremental cost per QALY (McIntosh and Luengo-Fernandez 2006). In the PT@3 trial, utilities and QALYs were estimated using a preference-based GHQoL instrument, the CHU9D.  Cost-effectiveness analysis (CEA) is an EE in which the effects of different interventions are measured using a single outcome, expressed as a natural unit (e.g. life years gained, reduction in d3mft, etc.). Alternative interventions are then compared in terms of incremental cost per unit of effect (e.g. incremental cost per unit reduction in d3mft) (McIntosh and Luengo-Fernandez 2006). In the PT@3 economic evaluation, CEA was conducted using the dental health effectiveness measure of d3mft. A “difference in difference” approach was used. The “d3mft difference” was used as an outcome, which for each child in each study group was the difference between their d3mft at 24-months minus their d3mft at 0-months.  Cost-consequence analysis (CCA) is a form of EE where disaggregated costs and a range of outcomes are presented to allow decision-makers to form their own opinion on relevance and relative importance to their decision making context (Drummond et al. 2005). This is usually done using a descriptive table to present the effectiveness results (both primary and secondary outcomes) in a disaggregated format, together with the estimates of the mean costs with appropriate measures of dispersion associated with each intervention (Hunter and Shearer 2019). In the case of CCA, all impacts and costs are considered (even if the impacts cannot be costed) when deciding which interventions represent the best value. This type of analysis provides a “balance sheet” of outcomes that decision-makers can weigh up against the costs of an intervention (NICE 2013).  In PT@3, the mean total costs and various outcome measures were compared. The outcome measures at 24 months were: d3mft, d3mft increment, OHQoL measures (namely, SOHO-5 and PedsQL-OH total scores and by item scores) and GHQoL measures (QALYs accumulated over the 24-month study period, utility index, the PedsQL total score and PedsQL domains, PedsQL and CHU9D scores by item). |
| --- |

References:

Drummond, M. F., M. J. Sculpher, G. W. Torrance, B. J. O'Brien and G. L. Stoddart (2005). Methods for the economic evaluation of health care programmes. Oxford, Oxford University Press.

Hunter, R. and J. Shearer. (2019). "Cost-consequences analysis - an underused method of economic evaluation." from <https://www.rds-london.nihr.ac.uk/resources/health-economics/cost-consequences-analysis-an-underused-method/>.

McIntosh, E. and R. Luengo-Fernandez (2006). "Economic evaluation. Part 2: frameworks for combining costs and benefits in health care." BMJ Sexual & Reproductive Health 32(3): 176.

NICE. (2013). "Guide to the methods of technology appraisal 2013. Process and methods." Retrieved August, 2020, from <https://www.nice.org.uk/process/pmg9/chapter/foreword>.

Appendix Figure 3: PT@3 staff cost form

**
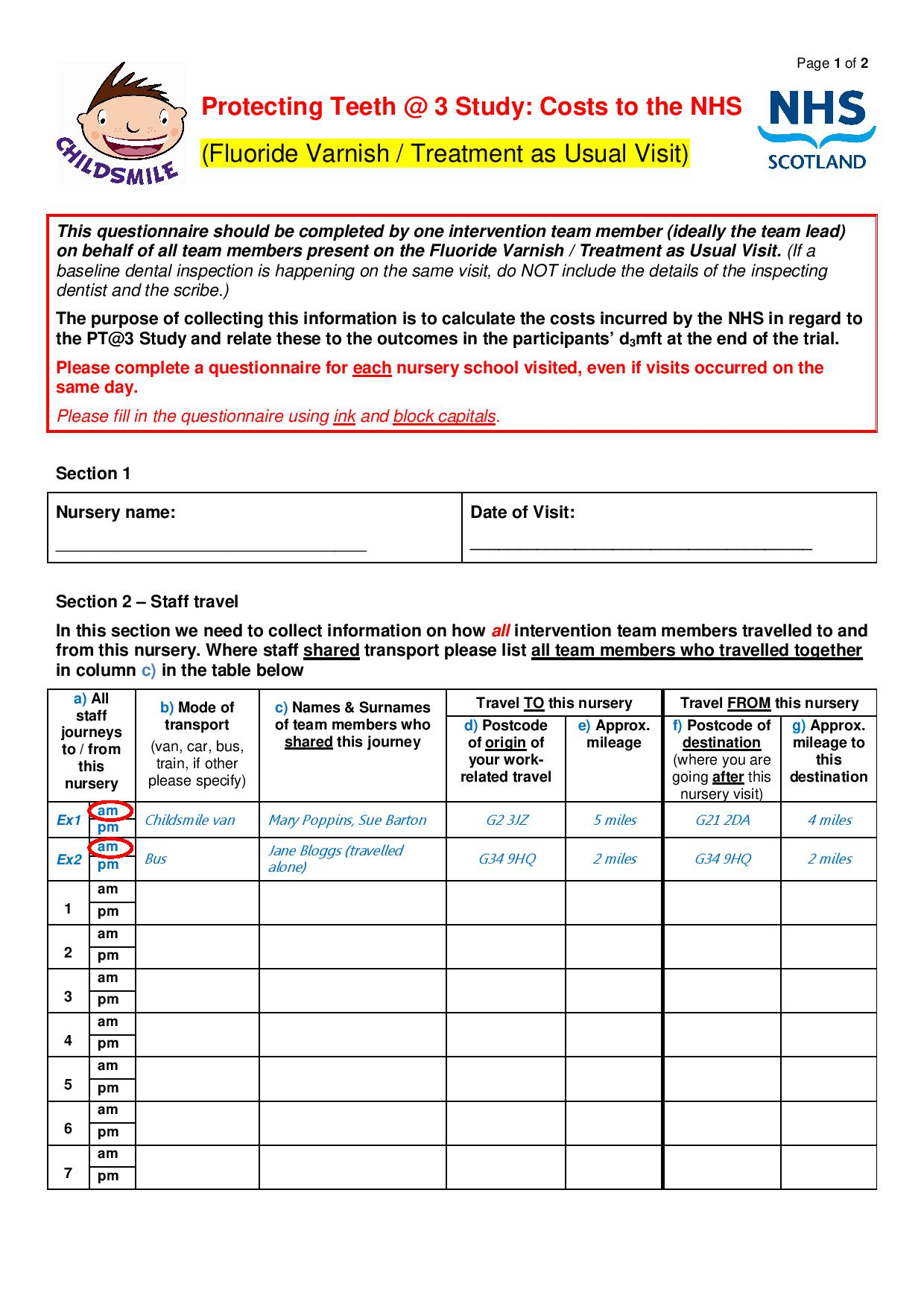
**

**
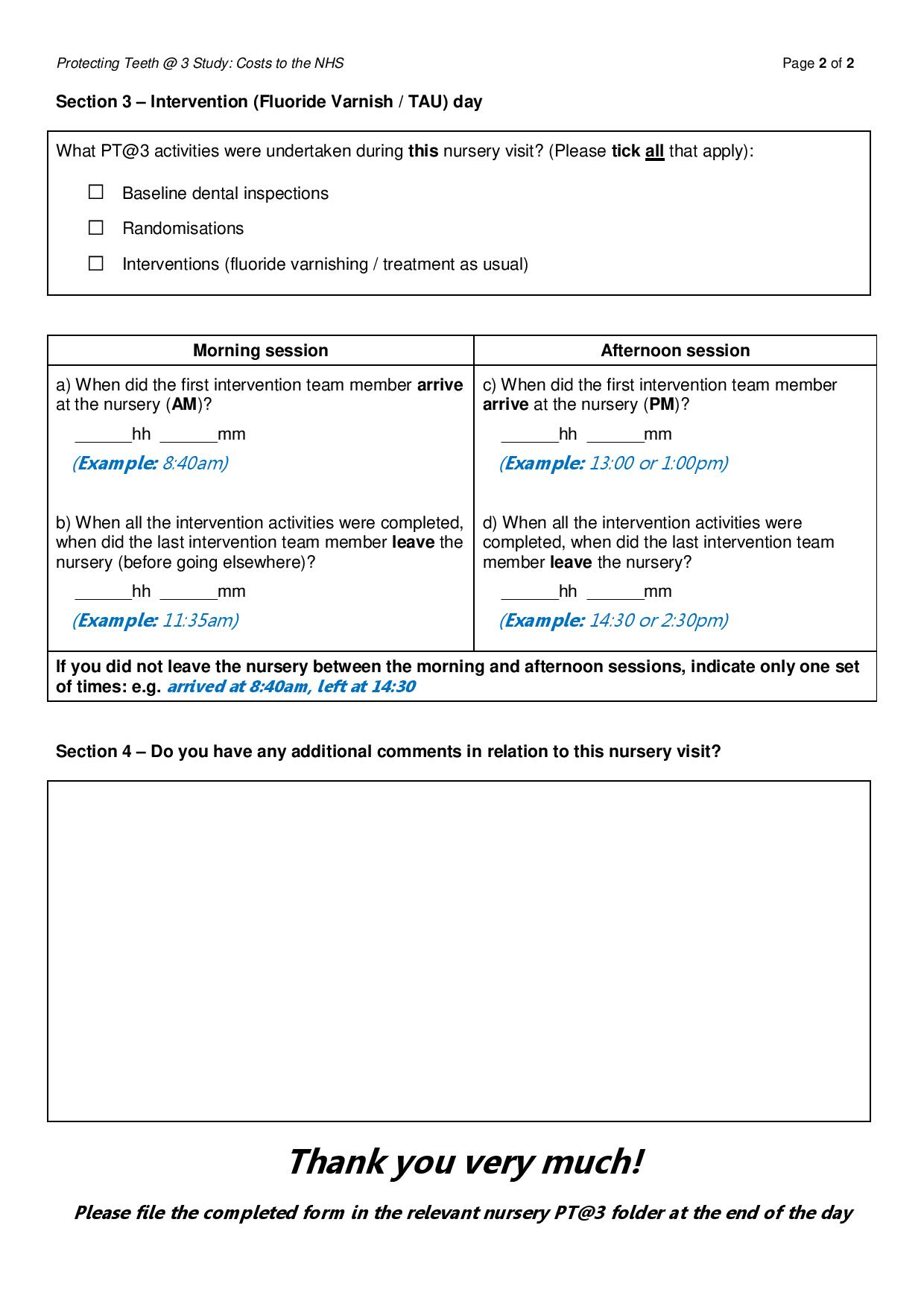
**

Appendix Table 2: Resource use unit costs, baseline year is 2016/17

| **Resource use item**  (per visit, unless otherwise stated) | **Unit cost** | **Source** |
| --- | --- | --- |
| General Practitioner  (GP) | £37 | PSSRU 2017 p. 162. Per patient contact lasting 9.22 minutes. |
| Accident and Emergency (A&E) attendance | £192.33 | NHS Reference costs 2016/17: Average of ‘see and treat and convey’ (by ambulance) (£248), ‘see and treat or refer’ (emergency care only) (£181) from Table 6, p. 9: ‘Costs by currency for ambulance services between 2014/15 and 2016/17’, and A&E attendance (£148); Table 2, p. 5: ‘Unit costs by point of delivery, 2014/15 to 2016/17’. |
| Dentist | (127+186) / 2 = £156.50  £156.50 x 0.167 = £26.13 | PSSRU 2017 p. 165-166. NHS dentist – Performer-only* £127 per hour of patient contact. NHS dentist – providing performer** £186 per hour of patient contact. Then the average of the two figures was taken.  Assumption: 10 min (which is 0.167 of an hour) for each appointment. |
| Dental hygienist | 1) £24.02 inflated from 2013/14 to 2016/17 is £25.00  2) Adding 55.1% earnings-to-expenses ratio = £38.77  3) £38.77 x 0.167 = £6.46 – cost of a 10 min appointment | British Dental Association (BDA) survey, ‘Dental Care Professionals’ Pay – Findings from the Dental Business Trends survey’, 2013 (Edwards 2013), p.16, Appendix III, Average pay table for dental hygienists (£/hour): £24.02, Scotland figure.  Inflated to 2016/17 level using HCHS annual pay indices – from PSSRU 2017 (p. 216)  A 55.1% earnings to expenses ratio (Scotland 2016/17 data) added (NHS Digital 2018), following the Northern Ireland Caries Prevention In Practice (NIC-PIP) trial methodology (Tickle et al. 2016).  Assumption: 10 min for each appointment.  Health and Social Care Information Centre / NHS Digital: Dental Earnings and Expenses Estimates, 2016/17, p. 118, Tab 23.1 (bottom line, All / All) |
| Speech and language therapist | £94.91 | NHS reference cost 2016/17 main schedule (Table ‘Total Other Currencies’): A13C1 ‘Speech and Language Therapist, Child, One to One’ |
| Hospital inpatient stay (per night) | £397.90 | NHS reference cost 2016/17 main schedule, ‘Total HRGs’ tab; ‘Regular day or night admissions’ – Column Y. Average over all paediatric services (from record PC63A until PX57C). |
| Hospital outpatient stay | £198.20 | NHS reference cost 2016/17 main schedule, Tab ‘Total Outpatient Attendances’, service code 420 – Paediatrics |
| Other resources filled in by parents (free text) | Various | Individually costed. |
| Medications used (free text) | - | Costs were not assigned.  We looked at whether any medication was used by each child (yes / no) to check if there was any difference between the groups. It was then considered that the use of medications was similar between the groups, and hence medications were not costed. |

Notes: * A performer-only dentist is a qualified dentist who works in a provider-performer practice (e.g. a local dental practice). They are sometimes referred to as Associates.

** A providing-performer, which is a dentist who holds a General Dental Services contract and/or a Personal Dentist Services agreement with the NHS. They also act as a performer, delivering dental services themselves.

References: PSSRU 2017 (Curtis and Burns 2017); NHS Reference costs 2016/17 (NHS Improvement 2017a); NHS reference cost 2016/17 main schedule (NHS Improvement 2017b)

Curtis, L. and A. Burns (2017). Unit Costs of Health and Social Care 2017. University of Kent, Canterbury, Personal Social Services Research Unit.

NHS Digital (2018). Dental Earnings and Expenses Estimates 2016/17.

NHS Improvement. (2017a). "2016/17 NHS reference costs and guidance." Retrieved July, 2019, from <https://improvement.nhs.uk/resources/reference-costs/#archive> .

NHS Improvement. (2017b). "Reference Cost Collection: National Schedule of Reference Costs, 2016-17 - NHS trusts and NHS foundation trusts." from <https://improvement.nhs.uk/resources/reference-costs/#archive>.

Tickle, M., C. O'Neill, M. Donaldson, S. Birch, S. Noble, S. Killough, L. Murphy, M. Greer, J. Brodison, R. Verghis and H. V. Worthington (2016). "A randomised controlled trial to measure the effects and costs of a dental caries prevention regime for young children attending primary care dental services: the Northern Ireland Caries Prevention In Practice (NIC-PIP) trial." Health Technology Assessment (Winchester, England) 20(71): 1-96.

Appendix Table 3: Economic evaluation resource use measures

| **Resource use category** | **Description of resource used** | **Unit of measure** |
| --- | --- | --- |
| **Intervention costs** | |  |
|  | Dental nurses’ time delivering PT@3 | Hours / minutes |
|  | Dental nurses’ travel related to delivering PT@3 | Mileage |
|  | Disposable items used per child (by study arm: intervention / control) | £ |
|  | Reusable items used, cost per child (across both study arms) | £ |
| **Participant healthcare resource use** | | |
|  | General Practitioner (GP) | No. of visits |
|  | Accident and Emergency (A&E) | No. of visits |
|  | Dentist | No. of visits |
|  | Dental hygienist | No. of visits |
|  | Speech and language therapist | No. of visits |
|  | Hospital inpatient stay | No. of nights |
|  | Hospital outpatient stay | No. of visits |
|  | Other resources – filled in by parents/carers (free text) | No. of visits / Other (depending on the nature of a healthcare resource used) |
|  | Medications used (free text) | Dichotomized: any medication used / not used (Yes / No) |
| **Family** |  |  |
|  | Time away from work / usual activities (due to child’s ill health) | Days |

Appendix Box 2: Handling missing data

| The data were considered missing if participants were still in the study at each point in time (had not withdrawn from the study) but did not have certain costs or utilities available in the study database.  Missing health and dental care resource use costs were treated differently, depending on the pattern of missingness in each returned parental questionnaire. If the questionnaire was not returned altogether (at any of the three distribution points) or a whole resource use section was left blank by the respondent, these resource use costs were considered to be missing. However, in the case where a respondent put some ineligible information in a resource use field, it was assumed that the child did use that service and a mean number of contacts based on all available cases per each round of questionnaire distribution (at 0, 12 or 24 months) was assigned.  The remaining missing baseline (0-month) resource use and utility data were imputed using mean imputation (the mean of all values observed at baseline, by item, was used) (Faria et al. 2014). It ensures that the imputed values are independent of the treatment allocation.  Multiple imputation (MI) with chained equations (Faria et al. 2014, Paton et al. 2016) was used to handle missing data on resource use costs and outcomes at the 12- and 24-months points.  The predictive mean matching method for multiple imputation was used to account for the non-normality of the distribution of costs and utility scores. This method ensures that the imputations took only values from the data that were available in the original trial data (Paton et al. 2016). By applying predictive mean matching, predictions that lie outside the bounds of each variable were avoided (White et al. 2011).  The resource use costs were imputed at the total resource use cost level (i.e. all resource use items summed up by participant at each questionnaire round), and the missing CHU9D data were imputed at a utility score level. This was done to avoid convergence issues of imputation model when containing many variables. It was assumed that data were missing at random. Intervention and control groups were imputed separately (Faria et al. 2014). A total of 50 imputed data sets was generated to improve efficiency (Graham et al. 2007, White et al. 2011). To further inform the imputation model the following auxiliary variables were included: age, sex, level of deprivation (SIMD), caries at baseline, baseline utility, baseline parental time off work and baseline child time off nursery.  Cost data often have a heavily zero-inflated right skewed distribution (Glick et al. 2014), and as the PT@3 children were a generally healthy population, the predictive mean matching method on log-transformed costs was employed as recommended in this case (MacNeil Vroomen et al. 2016). A £1 constant was added to the raw cost data to avoid problems when transforming zero values (Glick et al. 2014) before the log-transformation. The distribution of utilities in generally healthy populations is usually left skewed, with most children reporting high GHQoL (closer to 1) and smaller numbers in worse health states, therefore a similar manipulation was done with the utility values. First, a constant was introduced (1.1 minus the utility value), and then these resulting values were log-transformed. After imputation, both the cost and utility data were transformed back to the original scale for estimation (MacNeil Vroomen et al. 2016).  Following the use of MI, the uncertainty of the generated values was incorporated in the estimation of mean costs and utilities using a Rubin’s rule (Rubin 2004, Paton et al. 2016). |
| --- |

References:

Faria, R., M. Gomes, D. Epstein and I. R. White (2014). "A Guide to Handling Missing Data in Cost-Effectiveness Analysis Conducted Within Randomised Controlled Trials." PharmacoEconomics 32(12): 1157-1170.

Glick, H. A., J. A. Doshi, S. S. Sonnad and D. Polsky (2014). Economic evaluation in clinical trials, OUP Oxford.

Graham, J. W., A. E. Olchowski and T. D. Gilreath (2007). "How Many Imputations are Really Needed? Some Practical Clarifications of Multiple Imputation Theory." Prevention Science 8(3): 206-213.

MacNeil Vroomen, J., I. Eekhout, M. G. Dijkgraaf, H. van Hout, S. E. de Rooij, M. W. Heymans and J. E. Bosmans (2016). "Multiple imputation strategies for zero-inflated cost data in economic evaluations: which method works best?" The European Journal of Health Economics 17(8): 939-950.

Paton, N. I., W. Stohr, L. Oddershede, A. Arenas-Pinto, S. Walker, M. Sculpher and D. T. Dunn (2016). "The Protease Inhibitor Monotherapy Versus Ongoing Triple Therapy (PIVOT) trial: a randomised controlled trial of a protease inhibitor monotherapy strategy for long-term management of human immunodeficiency virus infection." Health Technol Assess 20(21): 1-158.

Rubin, D. B. (2004). Multiple Imputation for Nonresponse in Surveys, John Wiley & Sons.

White, I. R., P. Royston and A. M. Wood (2011). "Multiple imputation using chained equations: Issues and guidance for practice." Statistics in Medicine 30(4): 377-399.

Appendix Table 4: Sensitivity analyses scenarios investigated

| **Sensitivity analysis** | **Element** | **Variation for the sensitivity analysis** |
| --- | --- | --- |
| SA1 | Costs | Intervention costs (related to the “mock” applications) were added for TAU children. |
| SA2 | Costs | “Other” resource use costs added for both FV and TAU groups (i.e. the costs of the healthcare resources listed by the respondents in a free text field under “other” resources used) |
| SA3 | Costs | Intervention costs 30% less than in the baseline scenario. |
| SA4 | Costs | Intervention costs 30% greater than in the baseline scenario. |
| SA5 | Discount rate | Use of a traditional 3.5% discount rate for costs and outcomes |
| SA6 | Perspective / Costs | Cost of parental time off work (due to child’s health issues) was added. This represents societal costs perspective. |
| SA7 | Missing data | Available case analysis. Data assumed to be missing completely at random. |
| SA8 | Outliers (participant healthcare resource use / cost) | Four outlier observations were removed from the dataset (those children who had more than 45 Speech and Language Therapist contacts indicated at either 12-mo. or 24-mo. data collection points). |

In Scenario SA1 intervention costs (related to the “mock” application) were added for TAU children. This scenario reflects the actual PT@3 trial logistics, when the PT@3 dental nurse teams did see the children from the control arm, but instead of the real fluoride varnish being applied to their teeth, their teeth were touched with an empty applicator (without an active ingredient). In the case of the control group children, the intervention cost would include the cost of labour, cost of travel to/from the nursery (labour and travel costs were the same per child as for the intervention group children by individual nursery visit), cost of disposables (without the fluoride varnish cost) and cost of reusables (same as for the intervention group children).

Scenario SA8 was added post-hoc, when outlier observations in relation to participant healthcare resource use were identified. These were the four children who had more than 45 Speech and Language Therapist contacts indicated at either the 12-months or 24-months data collection points, who consequently had substantially higher healthcare resource use costs than the rest of the sample. In Scenario SA8 these four observations were removed from the dataset.

Appendix Table 5: Cost-effectiveness analysis methods

| Cost-effectiveness analysis (CEA) was conducted using the dental health effectiveness measure of d3mft. The d3mft effectiveness data were analysed using a “difference in difference” approach. The “d3mft difference” was used as an outcome, which for each child in each study group was the difference between their d3mft at 24-months minus their d3mft at 0-months. A positive d3mft difference means worsening of the oral health state. The principle used to calculate the ICER is shown in the table below. Here, the difference in difference is [(e-c) - (f-d)].  ICER for cost-effectiveness analysis on d3mft   \|  \| **Total cost (mean)** \| **d3mft at baseline (mean)** \| **d3mft at end of study (mean)** \| **Difference in d3mft** \| **ICER** \| \| --- \| --- \| --- \| --- \| --- \| --- \| \| **FV** \| a \| c \| e \| (e-c) \| (a-b) / ((e-c) - (f-d)) \| \| **TAU** \| b \| d \| f \| (f-d) \|  \| \| **Difference** \| (a-b) \|  \|  \| (e-c) - (f-d) \|  \| |
| --- | --- | --- | --- | --- | --- | --- | --- | --- | --- | --- | --- | --- | --- | --- | --- | --- | --- | --- | --- | --- | --- | --- | --- | --- |

Appendix Table 6: Cost-consequence analysis methods

| The components of the cost-consequence analysis (CCA) are presented in the table below. The mean values by group with the corresponding 95% confidence intervals (CI) were reported, as well as the mean difference between the groups with 95%CI. Total costs and various outcome measures were compared. The outcome measures at 24 months were: d3mft, d3mft increment, OHQoL measures (namely, SOHO-5 and PedsQL-OH total scores and by item scores) and GHQoL measures (QALYs accumulated over the 24-month study period, utility index, the PedsQL total score and PedsQL domains – physical, emotional, social, school and psychosocial).  Total costs and accumulated QALYs were analysed based on the multiple-imputed dataset, whereas d3mft, utility, OHQoL and GHQoL measures were analysed based on the available cases (i.e. the missing values were not imputed).  Cost-consequence analysis components   \| **Costs / Outcomes** \| \|  \| \|  \| \| \|  \| \| --- \| --- \| --- \| --- \| --- \| --- \| --- \| --- \| \| **Costs (MI dataset)** \| \|  \| \|  \| \| \|  \| \| Total cost \| \|  \| \|  \| \| \|  \| \| **Outcomes:** \| \|  \| \|  \| \| \|  \| \| QALY \| \|  \| \|  \| \| \|  \| \| **Dental health (ACA)** \|  \| \|  \| \|  \| \| d3mft at 0 mo. \| \|  \| \|  \| \| \|  \| \| d3mft at 24 mo. \| \|  \| \|  \| \| \|  \| \| d3mft difference (d3mft at 24 mo. minus d3mft at 0 mo.) \| \|  \| \|  \| \| \|  \| \| **OHQoL outcomes at 24 mo. (ACA)** \| \| \| \| \| \| PedsQL-OH score \| \|  \| \|  \| \| \|  \| \| SOHO-5 score \| \|  \| \|  \| \| \|  \| \| **GHQoL outcomes at 24 mo. (ACA)** \| \| \| \| \| \| Utility (CHU9D) \| \|  \| \|  \| \| \|  \| \| PedsQL - Total score \| \|  \| \|  \| \| \|  \| \| PedsQL - Physical domain score \| \|  \| \|  \| \| \|  \| \| PedsQL - Emotional domain score \| \|  \| \|  \| \| \|  \| \| PedsQL - Social domain score \| \|  \| \|  \| \| \|  \| \| PedsQL - School domain score \| \|  \| \|  \| \| \|  \| \| PedsQL - Psycho-social domain \| \|  \| \|  \| \| \|  \| \|  \| \|  \| \|  \| \| \|  \| |
| --- | --- | --- | --- | --- | --- | --- | --- | --- | --- | --- | --- | --- | --- | --- | --- | --- | --- | --- | --- | --- | --- | --- | --- | --- | --- | --- | --- | --- | --- | --- | --- | --- | --- | --- | --- | --- | --- | --- | --- | --- | --- | --- | --- | --- | --- | --- | --- | --- | --- | --- | --- | --- | --- | --- | --- | --- | --- | --- | --- | --- | --- | --- | --- | --- | --- | --- | --- | --- | --- | --- | --- | --- | --- | --- | --- | --- | --- | --- | --- | --- | --- | --- | --- | --- | --- | --- | --- | --- | --- | --- | --- | --- | --- | --- | --- | --- | --- | --- | --- | --- | --- | --- | --- | --- | --- | --- | --- | --- | --- | --- | --- | --- | --- | --- | --- | --- | --- | --- | --- | --- | --- | --- | --- | --- | --- | --- | --- | --- | --- | --- | --- | --- | --- | --- | --- | --- | --- | --- | --- | --- | --- | --- | --- | --- | --- | --- | --- | --- | --- | --- | --- | --- | --- | --- | --- | --- | --- | --- | --- | --- |

**Appendix Table 7: CHEERS checklist, items to include when reporting economic evaluations of health interventions**

| **Section/item** | **Item No** | **Recommendation** | **Reported**  **on page No** |
| --- | --- | --- | --- |
| **Title and abstract** | | | |
| Title | 1 | Identify the study as an economic evaluation or use more specific terms such as “cost-effectiveness analysis”, and describe the interventions compared. | Title page (p.i, *“economic evaluation”*) |
| Abstract | 2 | Provide a structured summary of objectives, perspective, setting, methods (including study design and inputs), results (including base case and uncertainty analyses), and conclusions. | Abstract (p. i) |
| **Introduction** | | | |
| Background and objectives | 3 | Provide an explicit statement of the broader context for the study. | p.1 |
|  |  | Present the study question and its relevance for health policy or practice decisions. | p.2 |
| **Methods** | | | |
| Target population and subgroups | 4 | Describe characteristics of the base case population and subgroups analysed, including why they were chosen. | pp. 2-3 |
| Setting and location | 5 | State relevant aspects of the system(s) in which the decision(s) need(s) to be made. | pp. 2-3 |
| Study perspective | 6 | Describe the perspective of the study and relate this to the costs being evaluated. | p. 3  (a public sector perspective was taken, that of the UK’s NHS) |
| Comparators | 7 | Describe the interventions or strategies being compared and state why they were chosen. | p. 2 |
| Time horizon | 8 | State the time horizon(s) over which costs and consequences are being evaluated and say why appropriate. | p. 3  (The time horizon was the follow-up of the PT@3 Study: two years.) |
| Discount rate | 9 | Report the choice of discount rate(s) used for costs and outcomes and say why appropriate. | p. 3  (a discount rate of 1.5% was employed) |
| Choice of health outcomes | 10 | Describe what outcomes were used as the measure(s) of benefit in the evaluation and their relevance for the type of analysis performed. | p. 3 |
| Measurement of effectiveness | 11a | *Single study-based estimates:*Describe fully the design features of the single effectiveness study and why the single study was a sufficient source of clinical effectiveness data. | pp. 2-3,  and referenced previously published trial effectiveness paper (McMahon et al. 2020). |
|  | 11b | *Synthesis-based estimates*: Describe fully the methods used for identification of included studies and synthesis of clinical effectiveness data. | N/A |
| Measurement and valuation of preference-based outcomes | 12 | If applicable, describe the population and methods used to elicit preferences for outcomes. | Measurement – p. 3  (CHU9D) |
| Estimating resources and costs | 13a | *Single study-based economic evaluation:* Describe approaches used to estimate resource use associated with the alternative interventions. Describe primary or secondary research methods for valuing each resource item in terms of its unit cost. Describe any adjustments made to approximate to opportunity costs. | pp. 3-4,  Appendix Table 2 |
|  | 13b | *Model-based economic evaluation:*Describe approaches and data sources used to estimate resource use associated with model health states. Describe primary or secondary research methods for valuing each resource item in terms of its unit cost. Describe any adjustments made to approximate to opportunity costs. | N/A |
| Currency, price date, and conversion | 14 | Report the dates of the estimated resource quantities and unit costs. Describe methods for adjusting estimated unit costs to the year of reported costs if necessary. Describe methods for converting costs into a common currency base and the exchange rate. | p. 3  (2016/17 was used as the cost baseline year. All costs were valued in UK pounds sterling.) |
| Choice of model | 15 | Describe and give reasons for the specific type of decision-analytical model used. Providing a figure to show model structure is strongly recommended. | N/A |
| Assumptions | 16 | Describe all structural or other assumptions underpinning the decision-analytical model. | N/A |
| Analytical methods | 17 | Describe all analytical methods supporting the evaluation. This could include methods for dealing with skewed, missing, or censored data; extrapolation methods; methods for pooling data; approaches to validate or make adjustments (such as half cycle corrections) to a model; and methods for handling population heterogeneity and uncertainty. | pp. 4-5,  Appendix Box 2, Appendix Tables 4-6 |
| **Results** | | | |
| Study parameters | 18 | Report the values, ranges, references, and, if used, probability distributions for all parameters. Report reasons or sources for distributions used to represent uncertainty where appropriate. Providing a table to show the input values is strongly recommended. | p. 5,  Appendix Table 8-9 |
| Incremental costs and outcomes | 19 | For each intervention, report mean values for the main categories of estimated costs and outcomes of interest, as well as mean differences between the comparator groups. If applicable, report incremental cost-effectiveness ratios. | p. 5,  Tables 1 & 2 |
| Characterising uncertainty | 20a | *Single study-based economic evaluation:* Describe the effects of sampling uncertainty for the estimated incremental cost and incremental effectiveness parameters, together with the impact of methodological assumptions (such as discount rate, study perspective). | p. 6,  Appendix Table 10 |
|  | 20b | *Model-based economic evaluation:*Describe the effects on the results of uncertainty for all input parameters, and uncertainty related to the structure of the model and assumptions. | N/A |
| Characterising heterogeneity | 21 | If applicable, report differences in costs, outcomes, or cost-effectiveness that can be explained by variations between subgroups of patients with different baseline characteristics or other observed variability in effects that are not reducible by more information. | p. 7  (*The EE was conducted on a relatively small sample, which did not allow for meaningful subgroup analyses…, as had been initially planned.*) |
| **Discussion** | | | |
| Study findings, limitations, generalisability, and current knowledge | 22 | Summarise key study findings and describe how they support the conclusions reached. Discuss limitations and the generalisability of the findings and how the findings fit with current knowledge. | pp. 6-7 |
| **Other** | | | |
| Source of funding | 23 | Describe how the study was funded and the role of the funder in the identification, design, conduct, and reporting of the analysis. Describe other non-monetary sources of support. | Acknowledgements section, p. 9 |
| Conflicts of interest | 24 | Describe any potential for conflict of interest of study contributors in accordance with journal policy. In the absence of a journal policy, we recommend authors comply with International Committee of Medical Journal Editors recommendations. | Declaration of Conflicting Interests section, p. 9. |

| The **ISPOR CHEERS Task Force Report**, *Consolidated Health Economic Evaluation Reporting Standards (CHEERS)—Explanation and Elaboration: A Report of the ISPOR Health Economic Evaluations Publication Guidelines Good Reporting Practices Task Force,* provides examples and further discussion of the 24-item CHEERS Checklist and the CHEERS Statement. It may be accessed via the *Value in Health* or via the ISPOR Health Economic Evaluation Publication Guidelines – CHEERS: Good Reporting Practices webpage: <http://www.ispor.org/TaskForces/EconomicPubGuidelines.asp> |
| --- |

Appendix Table 8: Baseline characteristics of the economic evaluation sample

| **Variable** | **FV (n = 265)** | | **TAU (n = 269)** | | **Total (N = 534)** | |
| --- | --- | --- | --- | --- | --- | --- |
|  | **Mean** | **(SD)** | **Mean** | **(SD)** | **Mean** | **(SD)** |
| **Age** | 3.52 | (0.24) | 3.54 | (0.24) | 3.53 | (0.24) |
|  | **n** | **(%)** | **n** | **(%)** | **N** | **(%)** |
| **Sex** |  |  |  |  |  |  |
| Female | 136 | (51%) | 145 | (54%) | 281 | (53%) |
| Male | 129 | (49%) | 124 | (46%) | 253 | (47%) |
| **SIMD** |  |  |  |  |  |  |
| 1 | 24 | (9%) | 16 | (6%) | 40 | (7%) |
| 2 | 89 | (34%) | 101 | (38%) | 190 | (36%) |
| 3 | 69 | (26%) | 80 | (30%) | 149 | (28%) |
| 4 | 52 | (20%) | 41 | (15%) | 93 | (17%) |
| 5 | 30 | (11%) | 31 | (12%) | 61 | (11%) |
| Unknown | 1 | (0%) | 0 | (0%) | 1 | (0%) |
| **Caries at baseline *** | **n = 264*** | | **n = 267*** | | **n = 531*** | |
| Yes | 37 | (14%) | 38 | (14%) | 75 | (14%) |
| No | 227 | (86%) | 229 | (86%) | 456 | (86%) |

Notes: FV – Fluoride Varnish treatment group; TAU – Treatment As Usual group; SIMD – Scottish Index of Multiple Deprivation (there was a small amount of missing data, 4 in each group)

* Baseline caries (d3mft) data were not available for three children (one in the FV and two in the TAU group) due to issues with the dental inspection forms.

Appendix Table 9: Materials costs (FV group only)

| **Item** | **Cost (£)** | **Notes** |
| --- | --- | --- |
| **Disposable items used per child in the FV (intervention) group** | | |
| Thin plastic tray | £0.60 | All disposable item costs were provided by a PT@3 study coordinator. |
| Plastic dental mirror | £0.25 |  |
| Plastic FV brush | £0.08 |  |
| Cotton wool roll x 4 | £0.02 |  |
| Duraphat – fluoride varnish | £0.62 |  |
| Gloves | £0.04 |  |
| Hand gel | £0.01 |  |
| Paper towel | £0.70 |  |
| Total | £2.32 |  |
| **Reusable items used during the intervention visit** | | |
| Beanbag | £89.98 | The cost was provided by a PT@3 coordinator. |
| Clear plastic stack box (to hold all disposable FV materials) | £34.62 | The cost was provided by a PT@3 coordinator. |
| Lockable container (black metal box with a lock) | £7.89 | For Duraphat tubes storage. The cost was provided by a PT@3 coordinator. |
| Daray dental examination light on tripod | £400 | [Cost sources: http://www.daray.co.uk/shop/lighting/examination/x100-led-mobile-examination-light.html (£420)   https://www.medisave.co.uk/daray-x100-led-examination-light-with-flexible-arm-mobile.html (£395)](file://\\campus.gla.ac.uk\SSD_Dept_Data_D\MVL\MVLPublic\MED\DentalSchool\DPHU\YuliaPhD\JDR%20paper\Cost%20sources:%20http:\www.daray.co.uk\shop\lighting\examination\x100-led-mobile-examination-light.html%20%20(£420)%20https:\www.medisave.co.uk\daray-x100-led-examination-light-with-flexible-arm-mobile.html%20(£395)) |
| Total reusable items cost | £532.49 |  |
| Total reusable items cost per child per visit | £0.08 | See the box below for calculation (Average attributed cost of reusable items per child per visit). |

**Average attributed cost of reusable items per child per visit:**

| In order to calculate the average attributed cost of reusable items per child per intervention visit, the equivalent annual cost (EAC) formula was used:   \| 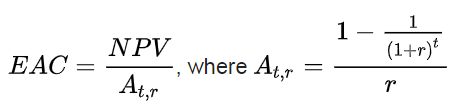  where *NPV* is the net present value, *r* is the discount rate and *t* is the number of years (in this case, an average life span of there years was used). \| \| --- \|   The net present value (NPV) of £532.49 (the total cost of all reusable items used during each intervention visit) was used, the discount rate (r) was 1.5% and the number of years (t) was three years – an average life span. The resulting EAC was £182.85. It was assumed then that each reusable ‘kit’ was used every working day by the usual mainstream Childsmile programme, which equated to five working days a week, resulting in 240 working days per year and, which, in turn, equated to the number of times each kit was used over a course of a year. The cost per each use was calculated as: EAC / 240 = £182.85 / 240 = £0.76. A mean cost per child per visit was then calculated: the average number of children seen per PT@3 intervention visit was ten, hence, the reusables cost per child, per visit was £0.76 / 10 = £0.076, which was rounded up to £0.08. |
| --- | --- |

Appendix Table 10: CUA sensitivity analyses results (after imputation) with 24-month follow-up

| **Analysis** | **Incremental cost** (Intervention minus Control) **(95% CI)** | **Incremental QALY** (Intervention minus Control) **(95% CI)** | **ICER**  **(£/ QALY gained)** | **Probability of being C-E at £20,000/QALY gained threshold (%)** |
| --- | --- | --- | --- | --- |
| **Base-case** | 68.37  (-18.04, 143.82) | -0.0044  (-0.016, 0.0069) | -15,467.26 | 11.3 |
| **SA1:** Intervention costs (of “mock” application delivery) added to TAU arm children | 36.64  (-48.51, 111.47) | -0.0044  (-0.016, 0.0069) | -8,288.66 | 17.1 |
| **SA2:** “Other” resource use costs included into total cost | 54.98  (-31.03, 130.76) | -0.0044  (-0.016, 0.0069) | -12,437.51 | 18.2 |
| **SA3:** Intervention costs are 30% less than in base-case | 58.11  (-26.34, 132.39) | -0.0044  (-0.016, 0.0069) | -13,145.57 | 12.5 |
| **SA4:** Intervention costs are 30% greater than in base-case | 78.60  (-7.17, 154.71) | -0.0044  (-0.016, 0.0069) | -17,780.01 | 9.2 |
| **SA5:** Discount rate of 3.5% | 67.27  (-36.78, 126.82) | -0.0044  (-0.016, 0.007) | -15,408.75 | 14.7 |
| **SA6:** Societal perspective –parental time off work included | 54.44  (-70.35, 167.84) | -0.0044  (-0.016, 0.0069) | -12,315.31 | 16.1 |
| **SA7:** Available case analysis | 73.23  (-31.60, 188.38) | -0.0078  (-0.2092, 0.0254) | -9,378.60 | 19.8 |
| **SA8:** Four outliers removed (with more than 45 Speech and Language Therapist contacts in a 12-mo. period) | 16.45  (-40.49, 75.70) | -0.0060  (-0.0197, 0.0056) | -2,736.05 | 13.7 |

Note: SA – sensitivity analysis. 1) General linear modelling was used. 2) Both cost and QALY were adjusted for sex, age, deprivation, baseline utility and caries at baseline. 3) Second year costs and QALYs were discounted at 1.5% (SA1-SA4, and SA6-SA8) or 3.5% discount rate (SA5).

Appendix Table 11: Results of cost-consequence analysis

| **Costs / Outcomes** | | **FV (intervention)** | | **TAU (control)** | | **Difference** |
| --- | --- | --- | --- | --- | --- | --- |
| **Costs (MI dataset)** | | **Mean (95% CI), £** | | **Mean (95% CI), £** | | **Mean (95% CI), £** |
| Total cost (base-case) | | 665.90  (564.38, 752.84)* | | 597.52  (519.29, 674.27)* | | 68.37  (-18.04, 143.82)* |
| Total cost, including “other” resources cost | | 674.16  (574.39, 764.51)* | | 619.18  (536.89, 701.59)* | | 54.98  (-31.03, 130.76)* |
| **Outcomes / Consequences** | | **Mean (95% CI)** | | **Mean (95% CI)** | | **Mean (95% CI)** |
| QALY (MI dataset) | | 1.8590  (1.8483, 1.8674)* | | 1.8634  (1.8522, 1.8729)* | | -0.0044  (-0.016, 0.0069)* |
| **Dental health (ACA)** |  | |  | |  | |
| d3mft at 0 mo. | | 0.428  (0.257, 0.599) | | 0.498  (0.298, 0.698) | | -0.07  (-0.333, 0.193) |
| d3mft at 24 mo. | | 1.371  (1.062, 1.681) | | 1.375  (1.029, 1.722) | | -0.004  (-0.467, 0.459) |
| d3mft difference, unadjusted  (d3mft at 24 mo. minus d3mft at 0 mo.) | | 1.016  (0.767, 1.264) | | 0.913  (0.678, 1.148) | | 0.103  (-0.239, 0.444) |
| d3mft difference, adjusted  (d3mft at 24 mo. minus d3mft at 0 mo.) | | 0.992  (0.761, 1.239)* | | 0.921  (0.695, 1.148)* | | 0.071  (-0.237, 0.406)* |
|  | | **FV (intervention)** | | **TAU (control)** | | **Difference** |
|  | | **Mean (95% CI)** | | **Mean (95% CI)** | | **Mean (95% CI)** |
| **OHQoL outcomes at 24 mo. (ACA)** | | | | | | |
| PedsQL-OH score | | 95.4  (93.7, 97.1) | | 95.5  (94.2, 96.7) | | -0.1  (-2.2, 2.0) |
| SOHO-5 score | | 0.3  (0.2, 0.4) | | 0.4  (0.2, 0.6) | | -0.1  (-0.3, 0.1) |
| **GHQoL outcomes at 24 mo. (ACA)** | | | | | | |
| Utility (CHU9D) | | 0.942  (0.932, 0.953) | | 0.946  (0.936, 0.956) | | -0.004  (-0.018, 0.01) |
| PedsQL - Total score | | 88.2  (86.5, 89.9) | | 88.7  (87.0, 90.4) | | -0.6  (-3.0, 1.9) |
| PedsQL - Physical domain score | | 92.1  (90.4, 93.8) | | 92.4  (90.4, 94.4) | | -0.3  (-2.9, 2.4) |
| PedsQL - Emotional domain score | | 79.8  (77.3, 82.4) | | 80.0  (77.5, 82.5) | | -0.2  (-3.7, 3.4) |
| PedsQL - Social domain score | | 88.6  (86.2, 91.0) | | 90.7  (88.6, 92.8) | | -2.1  (-5.3, 1.1) |
| PedsQL - School domain score | | 90.6  (88.6, 92.7) | | 90.2  (88.1, 92.3) | | 0.5  (-2.5, 3.4) |
| PedsQL - Psycho-social domain | | 85.7  (83.7, 87.6) | | 86.5  (84.6, 88.3) | | -0.8  (-3.5, 1.9) |

Notes: CI – confidence interval; MI – multiple-imputed; ACA – available case analysis; OHQoL – oral health related quality of life; GHQoL - general health related quality of life.

* Bootstrapped confidence intervals. QALY: the more QALYs are acquired the better. d3mft: the higher the value, the worse is the dental health. CHU9D utility values range from 0 (dead) to 1 (perfect health). PedsQL and PedsQL-OH: The higher the 0-100 scale score, the better is the quality of life. SOHO‑5: The lower the score, the better is the quality of life. The possible range is 0-28.

None of the cost or outcome differences between the two groups were statistically significant. In the base-case scenario, the mean total cost per child in the intervention group (which included intervention cost and participants’ NHS resource use costs) was £68.37 higher than in the control group. When “other” NHS resources costs were added to the total cost (as per the sensitivity analysis scenario SA2) this difference reduced to £54.98.

With regard to OH/GHQoL, the mean differences between the groups were mostly negative indicating that the children in the FV group had marginally worse OH/GHQoL at 24 months (although the differences were not statistically significant). The exceptions were the PedsQL school domain score where the difference was positive, and SOHO-5 score, where the negative difference means that the intervention group children had slightly better OHQoL (due to reversed scoring).

The FV group had marginally better dental health, i.e. lower mean d3mft, both at the baseline and at 24 months (again, the differences between the two groups were not statistically significant).
